# Supplementary material for: Long Term Immune Response Produced by the SputnikV Vaccine
Source: Int J Mol Sci. 2021 Oct 18;22(20):11211. doi: 10.3390/ijms222011211 (PMC8537212; doi:10.3390/ijms222011211)
Supplement: Supplementary file 1 [file ijms-22-11211-s001.zip › ijms-1382749-supplementary.pdf]

Table S1: Analysis of serum antibodies using Coronapass test.

Before vaccination (n=40)	0.27±0.41	
D21 after vaccination (n=40)	3.46±5.59	
D42 after vaccination (n=40)	10.72±5.43	
42 days* after COVID-19 (n=40)	6.55±4.83	
D0 before vaccination Vs D21 after vaccination	p-value	***0.00145****
D0 before vaccination Vs D42 after vaccination	p-value	***0.0001****
D0 before vaccination Vs 42 days* after COVID-19	p-value	***0.0001****
D21 after vaccination Vs 42 days* after COVID-19	p-value	***0.00072****
D42 after vaccination Vs 42 days* after COVID-19	p-value	***0.04735****
D21 after vaccination Vs 42 days* after vaccination	p-value	***0.0001****

* serum samples collected from convalescent COVID-19 between 32 and 65 days after convalescence (median 42±…days)

Table S2**:** List of currently circulating strains of SARS-CoV-2 used for the analysis

Accession	Strain	Collection Date
QUQ59779	B.1.1.1	25-04-2021
QUI75705	C.17	18-04-2021
QVI56963	B.1.617.2	22-04-2021
QSN65224	A.28	18-02-2021
QTH36725	B	30-03-2021
QUF59288	B.1	16-04-2021
QUW96083	B.1.1	15-04-2021
QTY73365	B.1.1.220	10-04-2021
QVT92231	B.1.1.306	01-04-2021
QVI39475	B.1.1.419	15-04-2021
QVI56975	B.1.1.7	15-04-2021
QUW05063	B.1.177	20-02-2021
QUF59095	B.1.190	12-04-2021
QUE44675	B.1.2	03-01-2021
QVQ62981	B.1.351	05-05-2021
QVT92411	B.1.36	01-04-2021
QVQ64625	B.1.36.27	16-04-2021
QUW05028	B.1.411	20-02-2021
QTY73401	B.1.466.2	10-04-2021
QTY73377	B.1.560	10-04-2021
QUF59047	B.1.617.1	12-04-2021
QSN65484	C.16	13-02-2021
QUF59107	C.36	12-04-2021
QUE44147	P.1	18-02-2021
BCU66482	R.1	2021-01
QTE07283	B.1.1.420	22-03-2021
QTD79351	B.1.177.75	22-03-2021
QTK04749	B.1.525	15-03-2021
QTT01560	B.1.160	01-03-2021
QTT38476	B.1.177.86	29-03-2021
QTT38032	B.1.258	06-03-2021
QTT37997	B.1.258.17	04-03-2021
QVP16947	A.2.5.1	03-05-2021
QVE07594	A.23.1	2021-04
QVM41414	B.1.1.222	01-05-2021
QVO27213	B.1.1.372	03-05-2021
QVO26892	B.1.241	01-05-2021
QVL88822	B.1.243	04-05-2021
QVN33214	B.1.311	08-05-2021
QVM53205	B.1.36.21	04-05-2021
QVO66036	B.1.375	11-05-2021
QVO20283	B.1.426	08-05-2021
QVM36334	B.1.429	01-05-2021
QVF80859	B.1.438.1	30-04-2021
QVO44234	B.1.526	08-05-2021
QVN38904	B.1.568	09-05-2021
QVM36370	B.1.575	01-05-2021
QVO41628	B.1.596	07-05-2021
QVP15844	B.1.612	02-05-2021
QVO34968	B.1.621	07-05-2021
QVN37384	B.1.623	10-05-2021
QVE49784	C.37	01-05-2021

Table S3: Unique mutations in S protein in SARS-CoV-2 strains selected for the analysis.

No	Peptide	Position	Mutation	Strain	Frequency	No	Peptide	Position	Mutation	Strain	Frequency
1		5	L>F	B.1.1.420, B.1.2, B.1.36.21, B.1.426, B.1.241, B.1.1.372, B.1.526, B.1.375, B.1.612	0.17	23	S9	152	W>L, W>R, W>C	R.1, C.17, B.1.429, B.1.575	0.08
2		12	S>F	B.1.190, C.36	0.04	24	S9	153	M>T	B.1.525	0.02
3		13	S>I	B.1.429	0.02	25	S9, S10	154	E>K	B.1.617.1	0.02
4		18	L>F	B.1.1.420, B.1.177.86, P.1, B.1.177	0.08	26	S9, S10	156	E>G	B.1.617.2, B.1	0.04
5		19	T>R	B.1.617.2, B.1	0.04	27	S10	157	F>L, F>S	A.23.1, B.1.258.17, B.1.1.220, B.1.1, B.1.1.419, B.1.623	0.12
6		20	T>N	P.1	0.02	28	S11	189	L>F	B.1.258.17	0.02
7		22	T>I	B.1.36	0.02	29	S11	190	R>S	P.1	0.02
8		25	P>H	B.1.623	0.02	30		215	D>H, D>G	B.1.190, B.1.351	0.04
9		26	P>S	P.1	0.02	31		222	A>V	B.1.177.75, B.1.177.86, B.1, B.1.177	0.08
10		27	A>S	B.1.351	0.02	32	S15	242	L>F	C.37	0.02
11	S3	49	H>Y	B.1.1.420, B.1.1.220, B.1.1, B.1.1.419	0.08	33	S15	253	D>G	B.1.36.21, B.1.426, B.1.241, B.1.621, B.1.526, B.1.375, B.1.612	0.13
12	S3	52	C>R	B.1.525	0.02	34	S21	346	R>S, R>K	C.17, B.1.621	0.04
13		64	W>R	B.1.1.1	0.02	35		367	V>F	A.23.1	0.02
14		67	A>V	B.1.525	0.02	36	S23	382	V>S	B.1.2	0.02
15		75	G>R, G>V	B.1.466.2, C.37	0.04	37	S23	385	T>I	B.1.243	0.02
16		76	T>I	C.37	0.02	38	S24	401	V>L	B.1.525	0.02
17		80	D>G, D>A	B.1.623, B.1.351	0.04	39	S24, S25	410	I>V	C.16	0.02
18		95	T>I	B.1.617.1, B.1.36.21, B.1.426, B.1.241, B.1.1.372, B.1.621, B.1.526, B.1.375, B.1.612	0.17	40	S25	417	K>T, K>N	P.1, B.1.351	0.04
19		98	S>F	B.1.575	0.02	41		439	N>K	B.1.258.17, B.1.258, B.1.466.2	0.06
20	S9	138	D>Y	P.1, B.1.1.1	0.04	42		440	N>K	B.1.1.420	0.02
21	S9	142	G>D	B.1.617.2	0.02	43	S27	444	K>N	B.1.243, B.1.1.420	0.04
22	S9	144	Y>F	B.1.177.75	0.02	44	S27	452	L>R, L>Q	C.16, B.1.617.1, B.1.190, C.36, B.1.617.2, B.1, C.17, C.37, B.1.429, B.1.623, A.2.5.1	0.21

Table S3: Unique mutations in S protein in SARS-CoV-2 strains used for the analysis (continued)

No.	Peptide	Position	Mutation	Strain	Frequency	No.	Peptide	Mutation	Position	Strain	Frequency
45	S28, S29	477	S>N	B.1.243, B.1.160, B.1.36.21, B.1.241, B.1.1.372, B.1.526, B.1.612	0.13	67	S45	754	L>F	B.1.621	0.02
46	S28, S29	478	T>K	B.1.617.2, B.1	0.04	68	S46	769	G>V	R.1	0.02
47	S29	484	E>K, E>Q	B.1.525, R.1, P.1, B.1.617.1, B.1.1.1, B.1.426, B.1.621, B.1.375, B.1.351	0.17	69	S46	772	V>I	B.1.258.17	0.02
48	S29	490	F>S	C.37	0.02	70		859	T>N	C.37, B.1.623	0.04
49	S29, S30	494	S>P	B.1.575	0.02	71		888	F>L	B.1.525	0.02
50	S30	501	N>T, N>Y	A.28, B.1.1.220, P.1, B.1.1, B.1.1.419, B.1.1.7, B.1.621, B.1.351	0.15	72		899	A>S	C.17	0.02
51		558	K>N	B.1.311	0.02	73		922	L>F	B.1.160	0.02
52		570	A>D	B.1.1.7	0.02	74	S56	950	D>A, D>N, D>H	B.1.160, B.1.617.2, B.1, B.1.623	0.08
53	S37	613	Q>H	A.23.1	0.02	75		957	Q>R	B.1.621, B.1.612	0.04
54	S37	614	D>G	B.1.243, B.1.525, C.16, B.1.177.75, B.1.1.420, B.1.160, B.1.177.86, B.1.258.17, B.1.258, , B.1.1.220, B.1.560, B.1.466.2, R.1, P.1, B.1.2, B.1.617.1, B.1.190, C.36, B.1.617.2, B.1, C.17, B.1.1.1, B.1.411, B.1.1, C.37, B.1.438.1, B.1.1.419, B.1.1.7, B.1.36.21, B.1.429, B.1.575, B.1.1.222, B.1.311, B.1.623, B.1.568, B.1.426, B.1.241, B.1.1.372, B.1.621, B.1.596, B.1.526, B.1.375, B.1.612, A.2.5.1, B.1.351, B.1.36.27, B.1.1.306, B.1.36	0.92	76		982	S>A	B.1.411, B.1.1.7	0.04
55	S37	621	P>S	B.1.243	0.02	77		1027	T>I	P.1, B.1.241	0.04
56	S37	622	V>F	B.1.36	0.02	78		1071	C>H	B.1.617.1	0.02
57	S39	647	A>S	B.1.596	0.02	79		1087	A>S	B.1.36.27	0.02
58	S39	653	A>Y	B.1.438.1	0.02	80	S66	1118	D>H	B.1.1.7, B.1.241	0.04
59	S39	655	H>Y	A.28, P.1	0.04	81	S66	1124	G>C	B.1.351	0.02
60		675	Q>H	C.37, B.1.438.1	0.04	82	S69, S70	1176	V>F	P.1	0.02
61		677	Q>H, Q>P	B.1.525, B.1.190, C.36, B.1.596, B.1.612	0.10	83	S71	1196	S>F	B.1.160	0.02
62		679	N>K	B.1.560	0.02	84		1241	C>S	B.1.1.1	0.02
63		681	P>H, P>R	B.1.243, A.23.1, B.1.466.2, B.1.617.1, B.1.617.2, B.1, B.1.411, B.1.1.7, B.1.575, B.1.621, B.1.1.306	0.21	85	S74	1243	C>S	B.1.1.1	0.02
64	S42	701	A>V	B.1.36.21, B.1.426, B.1.1.372, B.1.526, B.1.375, B.1.351	0.12	86	S74	1259	D>Y	B.1.258	0.02
65	S42	716	T>I	B.1.243, B.1.1.7, B.1.575	0.06	87		1264	V>L	B.1	0.02
66		732	T>A	B.1.1.222	0.02						
